# In this issue of Current Oncology

**Published:** 2006-06

**Authors:** M. McLean

Promoting best gynecologic oncology practice is the subject of a manuscript from the Society of Gynecologic Oncologists of Canada. This manuscript is a descriptive report from a meeting that took place in Montreal involving members of various Canadians societies interested in the management of patients with gynecologic malignancies. Although this manuscript only partly deals with the objectives of the meeting and does not provide a clinical practice guideline report (to be reported later), this important paper may lead and stimulate other oncologic societies to embark on similar exercises.

This month’s issue also contains the second part of a manuscript discussing the merits of naturally occurring anti-angiogenetic herbal compounds and their potential relevance to clinical practice. Encouragement is offered to explore the potential (and highly complex) effects in humans through clinical research. These agents have multiple effects and, presumably, a potential for multiple benefits.

The Genitourinary Disease Site Group of the Cancer Care Ontario Program in Evidence-based Care undertook a tricky review of the value of maximal androgen blockade in the treatment of meta-static prostate cancer. In this issue, the authors note that many meta-analyses have been published and yet the magnitude of any benefit remains unclear. The reported benefits of these agents (including their small impact on survival) is discussed—together with the their potentially negative impact on quality of life.

In discussing the impact of the geometric uncertainties associated with intensity modulated radiotherapy (imrt) treatment of head-and-neck cancers, Ballivy and colleagues address the subject of variation in target volumes and normal tissues during this form of radiation therapy delivery. The issue is not only important and relevant for head-and neck imrt, but also for all of radiotherapy as image guidance becomes the norm.

